# Vulvar Merkel Carcinoma: A Case Report

**DOI:** 10.1155/2011/546972

**Published:** 2011-05-18

**Authors:** C. Iavazzo, M. Terzi, P. Arapantoni-Dadioti, V. Dertimas, G. Vorgias

**Affiliations:** ^1^Gynecological Unit, Metaxa Memorial Hospital, Peiraeus, 38, Seizani Street, Nea Ionia, 14231 Athens, Greece; ^2^Pathology Unit, Metaxa Memorial Hospital, Peiraeus, 38, Seizani Street, Nea Ionia, 14231 Athens, Greece

## Abstract

This is a new case of Merkel cell carcinoma of the vulva. It is a rare neuroendocrine carcinoma with an aggressive behavior. Because of its rarity in this location, it is not clear whether it behaves differently from the usual neuroendocrine carcinomas of the skin. A case of a 63-year-old patient with vulvar Merkel carcinoma is presented. The clinical presentation, microscopic and immunohistochemical features, and treatment are discussed.

## 1. Introduction

The Merkel cell was first described by the German histopathologist Merkel in 1875 [1]. Merkel cells are components of the basal layer of the epidermis and the follicular epithelium. They form clusters in areas of sensory perception, close to primary nerve endings [2]. Primary neuroendocrine (Merkel) carcinoma of the skin was first described by Toker in 1972 [3]. It has an epidermal origin [4]. Vulvar Merkel cell carcinoma is a very rare entity with aggressive behavior. 

## 2. Case

A 63-year-old woman presented with a tumor of the left labium of the vulva. The patient claimed pruritus treated with corticosteroid cream the last 6 months. The biopsy revealed a Merkel cell carcinoma of the vulva. The tumor was stained with endocrine markers and cytokeratins 7 and 20. The cytokeratin 20 staining had a perinuclear dot pattern characteristic for Merkel cell carcinoma. It was chromogranin A, synaptophysin, CK18, CD56, and somatostatin positive. It had high mitotic index (90–100 *κ*.*ο*.*π*) and large number of apoptotic cells. The C/T scan showed left regional (inguinal) node metastasis. The tumor was 9 cm and lied from the urethra up to the perineum and deep to the periosteum of the pubic symphysis. Inguinal lymph node metastasis (5 cm) was present at the time of the surgery. She was treated with radical vulvectomy. Radiation therapy followed to the pelvis, perineum, vulva, and inguinal regions. 

## 3. Discussion

Merkel cell carcinoma affects elderly Caucasians (97%) with fair skin [5, 6]. Etiologic role plays the UV radiation [4]. It should be mentioned that viral etiology is also implicated in the pathogenesis as the recently discovered Merkel cell polyoma virus was found to infect the lymphoid system [7–9]. The median age is 69–75 years [5, 6]. It is most commonly found on sun-exposed areas such as the head or the neck (50–60%) [10] and the extremities, but it may also occur in the trunk or the genitalia. Tumor locations are buttocks (43%), extremities (36%), head (7%), unknown (14%) [11]. Because of its rarity, it is not known whether this neoplasm behaves differently in the vulvar location from at other sites [12]. Less than twenty cases of vulvar Merkel carcinomas are reported [12–14]. Furthermore, a few cases of Merkel cell carcinoma of the Bartholin's gland are reported in the bibliography [15]. Histologically, the tumor is characterised by intradermal small cells with high mitotic index and frequent apoptosis. The immunohistochemistry is positive for cytokeratins, epithelial membrane antigen, neurofilaments, neuron-specific enolase, and chromogranin A. Electron microscopy could reveal intermediate filaments in a typical globular paranuclear arrangement [16]. Merkel cells are usually identified by cytokeratin 20 stains [17]. Staging evaluation includes C/T and recently PET scan [18]. At postmortem examination, it was found that pelvic lymph nodes, liver, and vertebral metastases are possible metastases of vulvar Merkel cell carcinoma [19]. The diagnosis is frequently delayed [20]. It usually presents with regional lymph node metastases [5]. The treatment guidelines include local excision of the primary tumor with adjuvant radiotherapy [5]. A 3 cm excision margin is advocated, including fascia wherever possible [6]. Recent data show that treatment with surgical excision and adjuvant locoregional radiotherapy experiences a better disease-free interval than surgery alone [10]. Moreover, the role of adjuvant chemotherapy is still controversial; regimens for small cell carcinoma of the lung are also used. The combination of cyclophosphamide, doxorubicin, and vincristine has an overall response rate of 75% versus 60% of the cisplatin or carboplatin plus etoposide scheme [18]. It usually gives early local recurrences [5]. According to Lonardo et al., recurrence occurs in 86% of stage I and 20% of stage II tumors [11]. 

In the bibliography, there are limited data regarding the aggressive behaviour and poor prognosis of the tumor with reported survival rates ranging from 31% at three years up to 74% at five years [21]. Merkel cell carcinoma of the vulva seems to have a more aggressive behaviour and poorer prognosis than at other sites [12, 22]. 
